# Cultural distances between home and host countries inspire sojourners to engage in intercultural exchange upon repatriation

**DOI:** 10.1038/s41598-023-44906-w

**Published:** 2023-10-16

**Authors:** Xi Zou, Dan J. Wang, Tim Wildschut, Constantine Sedikides, Dan Cable

**Affiliations:** 1 Nanyang Technological University, Singapore, Singapore; 2 Columbia University, New York, USA; 3 University of Southampton, Southampton, UK; 4 London Business School, London, UK

**Keywords:** Psychology, Human behaviour

## Abstract

We examine how cultural distance between sojourners’ country of origin and their host country influences their engagement in intercultural exchange upon return. One might expect intercultural exchange to be much harder between culturally-distant countries than culturally-close ones, given that the former vary more in norms or expected behaviors from one’s home country. Our novel theorizing, however, leads to precisely the opposite expectations. In particular, we hypothesized that cultural distance between the repatriates’ home and host countries would be positively associated with being inspired by the host culture. In turn, this heightened inspiration would predict an increased sharing of knowledge about the host culture upon returning home (intercultural exchange). We combined measurement-of-mediation (Study 1) and experimental-causal-chain (Studies 2–3) approaches to test and confirm these hypotheses in three large samples of repatriates. We first examined whether cultural distance predicted greater intercultural exchange via repatriates’ heightened inspiration (Study 1). We then tested the individual links in this postulated causal chain. In Study 2, a quasi-experiment, we examined the causal path from cultural distance to inspiration. In Study 3, we experimentally manipulated inspiration to test its causal effect on intercultural exchange. The findings advance theory and application around multicultural experience and intercultural exchange.

## Introduction

Up to 2021, there were about 87.5 million expatriates globally, growing in a compound annual rate of 5.8% since 2013^1^. As more professionals choose to work in foreign countries, greater numbers eventually return to their home countries^2^. Given the deep impact of international immersion, many repatriates are understandably eager to share their overseas experiences. Such sharing (i.e., intercultural exchange) can benefit not only employees, but also employers and communities^3^. The significance of intercultural exchange has been formally recognized by the U.S. Congress’ Fulbright-Hays Act (the Mutual Educational and Cultural Exchange Act of 1961), which aims to promote various activities that can “increase mutual understanding between people of two nations” through the creation of temporary work visas allowing foreign nationals to work in the U.S. for short-term periods.

To understand what motivates repatriates to engage in intercultural exchange (i.e., promoting contact between home and host countries by being a cultural ambassador), we draw on cross-cultural psychology. We examine how cultural distance between sojourners’ origin and their host country influences whether they engage in intercultural exchange upon return. One might assume that intercultural exchange is much harder between culturally-distant countries than culturally-close countries, given that the former varies more in norms or expected behaviors from one’s home country. Our novel theorizing, however, leads to precisely the opposite prediction. Our perspective assigns a central role to the motivational state of inspiration. Using three large samples of international sojourners who worked in foreign countries and then returned to their home countries, we hypothesize and show that greater cultural distance between repatriates’ home and host countries is positively associated with being inspired by the host country. In turn, being inspired by the host country increases repatriates’ likelihood of initiating intercultural exchange upon returning home.

Cultural distance, the degree to which cultural norms and values in one country are different from those in another country^4^, shapes the subjective experience of sojourners. In particular, the greater the cultural distance between home and host countries, the more stressful the acculturation experience can be^5–7^. Two theoretical views account for the negative influence of cultural distance on acculturation. The *cultural-knowledge-learning* view emphasizes the difficulty of mastering dissimilar cultural knowledge. Successful adaptation hinges on the depth and speed with which sojourners acquire knowledge of host cultures, because such knowledge promotes assimilative behavior and helps sojourners fit in^8^. Thus, cultural distance impedes acculturation by increasing the difficulty of acquiring culture-specific knowledge about norms and customs^9,10^. The *cultural-identity-development* view, on the other hand, emphasizes the development of multicultural self-concepts. Sojourners who identify with the host culture and integrate it into their self-concept evince better adaptation^5,11^. Given that the newly formed self-concept will be based on markedly different or incompatible cultures^12^, stressful experiences such as cultural cognitive dissonance^13,14^ are likely to ensue. These two views imply that, because greater cultural distance is associated with poorer acculturation to the host country, repatriates who resided in more culturally distant host countries will experience more difficulty acquiring culturally-specific knowledge^15^ and will be less inclined to share their negative experiences (e.g., regret, distress) upon returning home. After all, sharing negative experiences elicits social rejection and devaluation^16^.

Going beyond the cultural-knowledge-learning and cultural-identity-development views, we propose that a larger cultural distance between the home and host countries can make sojourners feel more inspired. We theorize that inspiration, a motivational state, can reveal the hidden benefit for repatriates who return home from a culturally-distant country. Inspiration is often triggered by experiences that move people away from their routines and help them become aware of possibilities for new ideas or plans^17,18^. According to Csikszentmihalyi^19^, people are often inspired by a discrepancy in their own expertise domain that becomes obvious when viewed from the perspective of another domain. We posit that living and working in a culturally-distant country is prototypical of such an experience. Specifically, immersion in a foreign culture involves trying new and challenging activities, which are the basis for inspirational experiences^20^. Greater cultural distance signals a higher discrepancy between two domains: experiences in a culturally-distant country are more likely to be diverse, unique, and different from the routines of one’s home country. Using Csikszentmihalyi’s^19^ framework, a culturally-close country reflects a low-discrepancy domain, whereas a culturally-distant country reflects a high-discrepancy domain. Thus, repatriates who are from a culturally-distant (vs. close) country should be more inspired by their host culture (Hypothesis 1).

Inspired individuals are approach oriented^17^, that is, motivated towards a positive target^21^. Also, they are prone to telling others about the source of their inspiration, because a critical consequence of inspiration is the transmission (e.g., expression, imitation, narration) of qualities exemplified in the evocative object^22^. As noted by Thrash and Elliot (^17^, p. 871), “Inspiration implies motivation, which is to say that it involves the energization and direction of behavior,” such that people are keen to express what has inspired them. Indeed, inspiration is positively associated with creativity and productivity in writing and poetry^22^. We therefore hypothesize that repatriates who are inspired by their host culture will be more likely (upon return to their home country) to engage in intercultural exchange behaviors, such as talking more frequently about their experiences in the host country and sharing more eagerly what they have learned in the host culture (Hypothesis 2). Following the logic of Hypotheses 1 and 2, we propose that repatriates who return home from a culturally-distant (vs. close) host country will be more likely to engage in intercultural exchange as a result of experiencing a stronger sense of inspiration in the culturally-distant host country (Hypothesis 3).

## Results

We tested our hypotheses in three studies, using diverse datasets and methods. In Study 1, we analyzed a sample of international teachers who first worked in the U.S. and then returned to their home countries. We then switched to an experimental-causal-chain approach^23^ to test the individual links in the postulated mediational chain. Study 2 was a quasi-experiment examining the causal path from cultural distance to inspiration in a sample of U.S. professionals who had worked in multiple countries overseas. In Study 3, we then experimentally manipulated inspiration to test its causal effect on intercultural exchange in a sample of U.K. repatriates.

### Study 1

The sample consisted of 957 international teachers who completed a placement in U.S. school districts. The placements were full-time, salaried positions that included structured professional development opportunities. Placements were arranged by a U.S. company that served as an intermediary between international teachers and U.S. school districts, and they were allocated following a competitive application process. Key selection criteria included applicants’ educational background and experience. The intermediary company provided participants’ background data: age, gender, ethnicity, and the years they started and finished working as teachers in the U.S. We measured inspiration and intercultural exchange through an online survey. We derived cultural distance scores between a respondent’s home country and the U.S. using Hofstede’s dimensions^24^. Specifically, we followed the Kogut-Singh approach^4^ to combine Hofstede’s dimensions into an overall index of cultural distance (for details, see Supplementary Material). In this sample, the country with the greatest cultural distance from the U.S. was Costa Rica (5.29), and the country with the smallest was Australia (0.39). The three most highly represented countries were Colombia (17.8% of respondents; cultural distance = 4.00), the U.K. (12.25% of respondents; cultural distance = 0.70), and Spain (8.1% of respondents; cultural distance = 2.69).

We present zero-order correlations in supplementary material Table S1. As hypothesized, cultural distance was positively correlated with inspiration (*r*[753] = 0.28, *p* < 0.001) and intercultural exchange (*r*[753] = 0.23, *p* < 0.001). Inspiration and intercultural exchange were also positively correlated (*r*[753] = 0.46, *p* < 0.001).

Next, we included the control variables in OLS regression analysis with clustered standard errors by participant’s home country (Table 1). Consistent with Hypothesis 1, teachers who returned home from a culturally-distant country reported greater inspiration (β = 0.137, *t* = 2.73, *p* = 0.010). Consistent with Hypothesis 2, inspiration predicted more intercultural exchange, above and beyond cultural distance and the control variables (β = 0.377, *t* = 9.00, *p* < 0.001). As a final step, we used the *lavaan* package in R to test the indirect effect (denoted *ab*) of cultural distance on intercultural exchange, via inspiration. Consistent with Hypothesis 3, the indirect effect via inspiration was significant, *ab* = 0.092, 95% CI = [0.067, 0.118]. Further, we tested an alternative mediation model (showing poorer fit to the hypothesized model) and replicated the results with additional controls in supplementary material.

**Table 1:** Predicting inspiration and intercultural exchange in study 1.

Predictor	Dependent variable					
	Model 1: inspiration			Model 2: intercultural exchange		
	β	*t*	*p*	β	*t*	*p*
Age	− 0.061	− 1.59	0.120	0.068	1.81	0.079
Sex (0 = man, 1 = woman)	− 0.044	− 1.46	0.153	− 0.069	− 1.98	0.056
Years since return home	− 0.012	− 0.35	0.730	− 0.072	− 2.04	0.048
Years abroad in U.S	0.009	0.21	0.837	0.046	1.37	0.178
Program completion	− 0.026	− 0.94	0.354	− 0.020	− 0.70	0.487
Extraversion	0.029	0.73	0.467	0.062	1.75	0.088
Emotional Stability	− 0.022	− 0.50	0.618	− 0.043	− 0.85	0.404
Conscientiousness	0.019	0.32	0.751	0.048	1.48	0.147
Agreeableness	0.125	2.10	0.043	0.184	4.48	< 0.001
Openness to Experience	0.041	1.13	0.267	− 0.010	− 0.24	0.815
Home country GDP per capita	− 0.236	− 2.76	0.009	− 0.086	− 0.89	0.381
Home country population	− 0.056	− 1.39	0.174	− 0.008	− 0.10	0.921
Home country immigrant population	0.079	1.04	0.303	0.127	2.64	0.012
Home country democracy index	0.020	0.38	0.710	0.003	0.05	0.959
**Cultural distance**	**0.137**	**2.73**	**0.010**	**0.086**	**1.78**	**0.084**
**Inspiration**				**0.377**	**9.00**	** < 0.001**

β values are standardized beta coefficients from an OLS regression analysis. *t* values are based on cluster standard errors (clustered by home country).

In summary, repatriates from a country that was more culturally distant from their own were more inspired by their U.S. experience, and, in turn, this inspiration motivated them to engage in more intense intercultural exchange. These findings remained significant, even when controlling for Big Five personality (and other variables; in supplementary material), casting doubt on self-selection as an alternative explanation. Nevertheless, we address this issue more directly in the next study.

### Study 2

We had three objectives in Study 2. First, we attempted to replicate conceptually Study 1 using a different sample: American repatriates who went abroad and then returned to the U.S. Second, we addressed the inherent limitations of cross-sectional designs. Ideally, a researcher would randomly assign participants to repatriate from a culturally-distant or culturally-close country, but the practical challenges of such an experiment are formidable. Instead, we opted for a quasi-experimental within-subjects design that involved recruiting U.S. participants who had resided in multiple foreign countries, and instructing them to report on their experiences in the most and least culturally-distant host country (in counterbalanced order). This design also conclusively addresses self-selection as an alternative explanation, because each participant serves as their own control.

Third, we took into consideration the notion, based on the cultural-knowledge-learning and cultural-identity-development views, that cultural distance should impede acculturation to the host country (henceforth, acculturation). Poor acculturation, in turn, should decrease intercultural exchange upon returning home. To examine the possibility that the benefits and drawbacks of cultural distance occur simultaneously and independently, we tested the effect of cultural distance on inspiration (benefit) and on acculturation (drawback). Additionally, we tested the indirect effect of cultural distance on intercultural exchange via, respectively, inspiration and acculturation.

The joint consideration of benefits and drawbacks of cultural distances has two intriguing conceptual/statistical corollaries. By controlling for drawbacks of cultural distance (i.e., reduced acculturation), its positive effect on intercultural exchange should be strengthened (i.e., suppression or inconsistent mediation;^25^). Also, the countervailing indirect effects via inspiration (positive) and acculturation (negative) could offset each other. Accordingly, we devised a data-analytic strategy that clarifies these processes.

We recruited 237 participants for payment through Qualtrics using the following criteria: (1) American citizens who were born and raised in the U.S.; (2) lived and worked in at least two foreign countries for over six months in each; (3) currently employed full-time in the U.S. Participants first reported up to four countries in which they had lived most recently for at least six months, providing the start and end dates. On average, they reported 2.27 countries (*SD* = 0.56), and 73 unique countries in total. Kogut-Singh cultural distance scores (4) ranged from 5.29 (Costa Rica) to 0.39 (Australia). The most frequently reported host country was the U.K. (*n* = 51). An algorithm identified the two countries with the largest and smallest cultural distance to the U.S. for each participant.

Next, participants recalled, in counterbalanced order, their experiences in these two host cultures (most distant vs. least distant to U.S. culture). This constituted our within-subjects manipulation of cultural distance. Specifically, participants listed four keywords relevant to their experience in the given host culture. Then, they immersed into the experience, described it, and reflected on how it made them feel. Lastly, they answered questions assessing their acculturation, inspiration, and intercultural exchange. They repeated this sequence for the second host culture. The study ended with questions about participants’ demographics (i.e., age, gender, ethnicity).

We present statistical comparisons between the most and least culturally-distant host country in Table 2. Consistent with Hypothesis 1, participants reported being more inspired by their experiences in the most (compared to least) culturally-distant host country. Yet, as per prior research, participants reported having experienced lower acculturation in the most (compared to least) culturally-distant host country. Thus, cultural distance had opposite effects on inspiration and acculturation: Whereas it increased the former, it decreased the latter. Consistent with Hypothesis 2, inspiration was positively associated with intercultural exchange (*r*[237] = 0.66, *p* < 0.001). Acculturation was also positively associated with intercultural exchange (*r*[237] = 0.46, *p* < 0.001). Furthermore, inspiration and acculturation were positively related (*r*[237] = 0.38, *p* < 0.001).

**Table 2:** Means and standard deviations for dependent variables as a function of cultural distance in study 2.

	Least distant host country		Most distant host country		Cultural distance effect	
	*M*	*SD*	*M*	*SD*	*t*	*p*
Inspiration	5.56	1.26	5.73	1.12	2.65	0.010
Acculturation	1.91	1.07	1.67	0.90	− 2.64	0.009
Intercultural exchange	6.19	1.23	6.26	1.09	0.63	0.523

Tabled positive emotion scores are based on a square root transformation of raw scores. We contrast-coded the cultural distance variable (− 1 = *least distant host country*, 1 = *most distant host country*). *t* values are based on two-way cluster standard errors (clustered by participant and host country).

**Figure 1:**
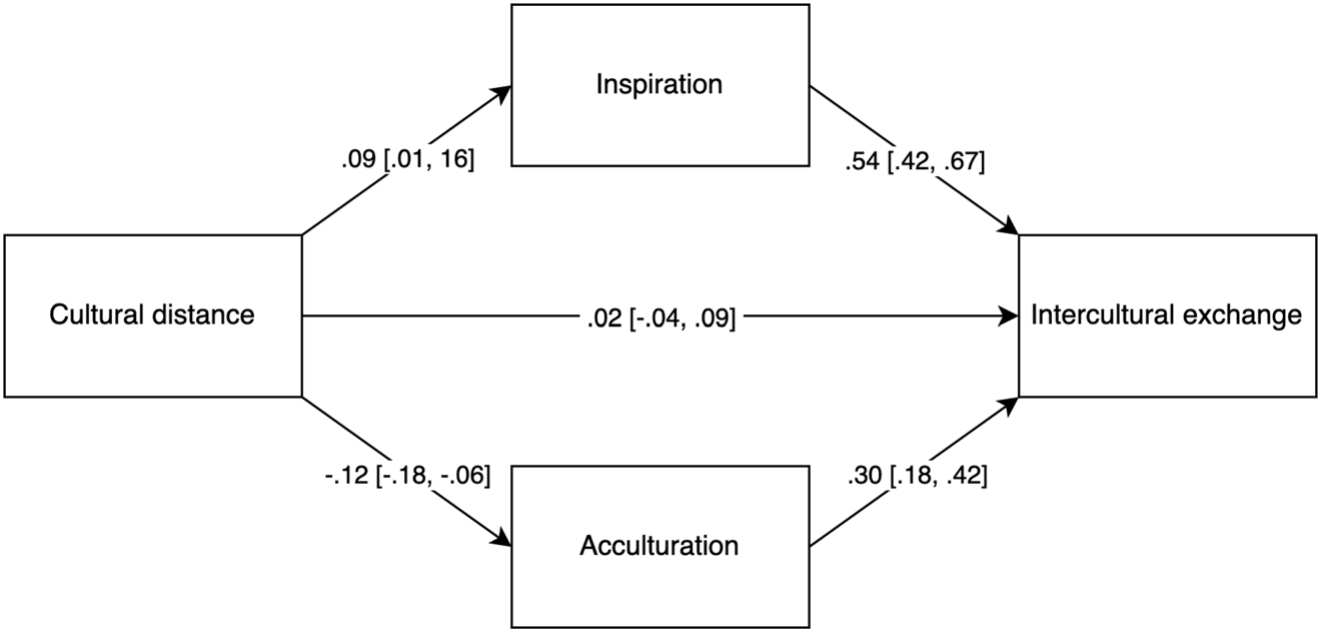
Opposing indirect effects of cultural distance on intercultural exchange via inspiration and acculturation in study 2. *Note.* Path coefficients are standardized. Numbers in parentheses indicate 95% confidence interval.

Next, we used the *lavaan* package in R to test the indirect effect of cultural distance on intercultural exchange via inspiration and acculturation, respectively. We modeled inspiration and acculturation as parallel mediators (Fig. 1). Supporting Hypothesis 3, the positive indirect effect via (increased) inspiration was significant, *ab* = 0.046, 95% CI = [0.005, 0.088]. The negative indirect effect via (reduced) acculturation was also significant, *ab* = − 0.036, 95% CI = [− 0.011, − 0.061]. Cultural distance impacted intercultural exchange via two countervailing pathways: a positive indirect effect via (increased) inspiration that replicated Study 2 findings, and a negative indirect effect via (decreased) acculturation. These two effect sizes do not differ significantly in absolute magnitude (*ab*_diff_ = 0.01, 95% CI = [− 0.045, 0.006]) but oppose in direction. This explains why the total effect of cultural distance on intercultural exchange was non-significant (Table 2).

In summary, Study 2 conceptually replicated the results of Study 1 and further attested to the generalizability of our findings. Cultural distance had the same effects regardless of whether respondents repatriated from the U.S. to various home countries or to the U.S. from various host countries. The implementation of a quasi-experimental within-subjects design provides a more stable underpinning for causal inferences, and conclusively ruled out self-selection as an alternative explanation because each participant served as their own control. Finally, we tested the indirect effect of cultural distance on intercultural exchange via both inspiration and acculturation, and demonstrated the simultaneous and independent operation of these countervailing processes.

### Study 3

We set three objectives in Study 3. First, Study 2 started the process of testing the postulated causal sequence by manipulating the independent variable (i.e., cultural distance). However, from the perspective of experimental-causal-chain design^23^, the mediator (i.e., inspiration) will need to be manipulated as well, to address the inherent limitations (i.e., reverse causation, third-variable problems) of cross-sectional measurement-of-mediation designs. Thus, we proceeded to manipulate inspiration and examine the causal effect of the mediator on the outcome.

Second, we constructed a behavioral measure of intercultural exchange. As in the preceding studies, we followed the conceptualization of intercultural exchange set forth by the Fulbright-Hays Act as activities that involve “sharing of experience of the host country” and can “increase mutual understanding between people of two nations.” Accordingly, we instructed participants to write an essay about their experiences in the host country. To index the extent to which participants acted as “de facto ambassadors and advocates [of the host country] abroad” (^3^, p. 17), coders rated the degree to which reading the essays made them want to visit the host country.

Finally, we tested British repatriates now living in the U.K., further examining the generalizability of our findings. We recruited 300 participants for payment through Prolific Academic using the following criteria: U.K. citizens who (1) were born and raised in the U.K.; (2) lived in a foreign country for over six months within the last five years; and (3) were currently employed full-time and living in the U.K.

Participants first listed a country in which they had lived for at least six months in the last five years, providing the start and end dates. They also reported information on the nature of their stay, and whether the international experience was self-initiated. In all, participants listed 57 countries. The most frequently listed host country was the U.S. (*n* = 34). On average, participants lived in the host culture for 1.80 (*SD* = 3.15) years and returned to the U.K. within the last 3.22 (*SD* = 1.99) years. International experience was more often self-initiated (79.76%, *n* = 239) than not self-initiated (20.33%, *n* = 61). However, self-initiation (yes vs. no) did not moderate the experimental effects, and thus we omitted this variable from the results below.

Next, we randomly assigned participants to one of two conditions: inspiration (*n* = 152) or control (*n* = 148)^18^. Subsequently, we instructed all participants: “Please write a short essay on things to do in [the host country]. For example, you can introduce places that you liked visiting the most or things that you enjoyed doing while you were in [the host country].” Finally, participants responded to the manipulation check, measures pertaining to control variables (positive affect, negative affect, effort), and the dependent variable (intercultural exchange). We used established scales to measure and recruited two coders to code the essays. Demographic questions (i.e., age, gender, ethnicity) concluded the study.

Participants in the inspiration condition (*M* = 6.14, *SD* = 0.70) reported higher levels of inspiration than those in the control condition (*M* = 5.34, *SD* = 1.34), *F*(1, 298) = 42.21, *p* < 0.001, ${\eta }_{p}^{2}$ = 0.12. The manipulation was effective. Further, positive affect (*F*[1, 298] = 0.04, *p* = 0.84, ${\eta }_{p}^{2}$ = 0.0001), negative affect (*F*[1, 298] = 2.10, *p* = 0.15, ${\eta }_{p}^{2}$ = 0.006), and writing effort (*F*[1, 298] = 0.09, *p* = 0.77, ${\eta }_{p}^{2}$ = 0.0003) did not differ significantly between conditions.

Supporting Hypothesis 2, essays in the inspiration condition (*M* = 2.64, *SD* = 0.72) were more likely to make coders want to visit the foreign country than essays in the control condition (*M* = 2.45, *SD* = 0.72), *F*(1, 298) = 5.52, *p* = 0.020, ${\upeta }_{\mathrm{p}}^{2}$ = 0.018. Inspiration increased intercultural exchange; that is, inspired repatriates are better “de facto ambassadors and advocates” of the host country (^3^, p. 17).

## Discussion

When sojourners live in another country, it changes them. To learn more about whether they bring their new ideas and perspectives back to their home country, we introduced and unpacked the psychological pathway of inspiration, through which experience in a distant culture contributes to intercultural exchange. Our findings suggest that sojourners from a home country that is more (vs. less) culturally distant from their host country are more likely to be inspired by their experience abroad. This inspiration, in turn, promotes their intercultural exchange upon returning home. The findings have implications for literatures on multicultural experience, intercultural exchange, and the management of human capital across borders.

Our findings help to clarify the role of cultural distance in the international adjustment process. The literature has focused mainly on the negative repercussions of cultural distance for acculturation, and few studies have considered the implications of cultural distance for repatriates. Our research redresses this imbalance by showing that cultural distance can confer benefits for repatriates and the organizations to which they return. We theorized and found evidence that cultural distance evoked greater inspiration among sojourners. Although inspiration is often triggered by experiences that move people away from their normal routines, the issue has been neglected in the literature on cultural distance and intercultural exchange. Upon returning home, repatriates have reaped valuable insights from their international and multicultural exposure. For example, living in more than one culture contributes to the development of a broader worldview^26^, stereotype de-biasing^27^, and creative performance^28,29^. The current findings add to this growing body of research by highlighting benefits of traversing cultural distances—in particular inspiration, a psychological mechanism that facilitates intercultural exchange.

By linking variation in cultural distance to individual-level outcomes, we built on work in international business that has used country distance measures—such as institutional distance^30^—to explain firm-level outcomes. For example, past cultural-distance research has addressed subsidiary location choice^31^, modes of entry and ownership^32,33^, and subsidiary performance and strategy^34,35^. The principal findings cohere around the notion that institutional or cultural distance increases a firm’s “liability of foreignness,” which can preclude access to local resources and information^36^. Most approaches at the firm level have emphasized the disadvantage that accompanies greater cultural distance. However, a meta-analysis indicated that the effects are not all negative: The linkage of cultural distance to firm-level outcomes was near zero across 66 independent samples^37^.

The present findings also contribute to the intercultural contact literature. The nature of intercultural interaction has evolved dramatically in the age of globalization. History is replete with examples of how intergroup contact leads to the enslavement, exploitation, or even murder of those who venture into the territory of a foreign ethnic group^38^. Nevertheless, intergroup contact can yield substantial gains in resources and knowledge through exchange or trade^39–41^. Our research suggests that intergroup contact can foster inspiration and intercultural exchange, which ultimately can benefit organizations and society more broadly. These benefits are realized to a greater extent by repatriates who have lived in a host country that is culturally distant from the home country.

Our investigation has several strengths, such as relying on diverse samples, implementing an experimental-causal-chain approach, and using both objective (i.e., cultural distance) and self-reported data. Our investigation also has certain limitations. In Study 1, we relied on a one-wave, cross-sectional survey design, potentially introducing self-selection bias. Alleviating these concerns, in Study 2 we conceptually replicated the Study 1 results ruling out selection bias by means of a within-subjects design in which each participant served as their own control. In Study 2, we also laid the foundation for stronger causal inferences with regard to the effects of cultural distance, yet future research would do well to complement quasi-experimental designs, such as the one implemented in Study 2, with longitudinal designs, which are better equipped to track the psychological journey of repatriates. Additionally, future research could attempt to assess intercultural exchange through behavioral indicators and peer ratings. Follow-up investigations might also examine the implications of cultural distance for various types of international professionals, such as flexpatriation, secondments, and globetrotting^42^.

Our theoretical perspective holds the implicit assumption that intercultural exchange is beneficial to society, organizations, and individuals in general. Indeed, this is the rationale underlying the U.S. Congress’ Fulbright-Hays Act to “increase mutual understanding between people of two nations.” Nevertheless, one cannot take for granted that intercultural exchange only entails benefits. Follow-up investigations could explore the potential downsides of intercultural exchange, such as the tendency toward Americanization and cultural hegemony^43^.

Our research focused on the psychological mechanism of the knowledge carrier; that is, their experience of inspiration. However, future investigations should also examine the recipients of intercultural knowledge. For example, these recipients might find the information and knowledge from a more (vs. less) distant culture to be more novel and interesting, thus making them more curious and open to intercultural exchange. Further, organizations in the home country can facilitate intercultural exchange by creating opportunities for interaction between knowledge carriers and potential recipients.

We recognize that cultural distance is not static. Cumulative data indicate that alterations in socioeconomic structure precede changes in individualism^44^. Recent evidence also suggests that, over the course of the twentieth century, individualism has increased in the U.S., manifesting itself in individualist book themes, uniqueness in baby names, and smaller family size^45^. We also recognize that our samples consist of fairly well-educated professionals (i.e., teachers, full-time employees) in developing countries. Future research should examine intercultural exchange among a wide range of professions, such as seasonal workers from Mexico or domestic workers from the Philippines. Although one might consider that our findings are restricted to cosmopolitan sojourners, an alternative possibility is that the positive association of cultural distance with inspiration is stronger among working-class international laborers as they lack alternative means, such as traveling or having foreign colleagues, of exposure to foreign cultures. For them, international exchange in a distant culture could truly be an eye-opening inspirational experience.

Future research would also need to explore the curvilinear effect of cultural distance. Here, we documented a positive linear association of cultural distance with pertinent outcome variables. Yet, moderate levels of cultural distance can be associated with the highest level of creative innovation^46,47^. That is, living and working in culturally similar host countries does not appear to provide the requisite novelty for creativity to emerge, whereas living and working in vastly dissimilar countries may engender high levels of distress, which may hinder creativity. Although we did not identify curvilinear effects in our studies, we cannot rule out the possibility that too much distance is associated with negative consequences. The absence of curvilinear effects might be due to the range of cultural distance factors or the type of outcome measures that we used.

A growing number of people move back and forth across national borders and cultural boundaries. Our studies revealed that repatriates returning from a culturally-distant host country were more inspired by their experience, which was positively associated with and increased intercultural exchange. We hope that our findings generate further research into the psychological dynamics of intercultural exchange.

## Methods

The protocol of following studies was reviewed and approved by the first author’s University Institutional Review Board (Reg. No. 200604393R; Title: The psychology of intercultural exchange). All methods were carried out in accordance and informed consent of all participants were obtained.

### Study 1

Of the 6,137 teachers who were invited via email to complete the online survey, 957 responded (15.6% response rate). There were no significant differences in terms of gender composition or ethnicity between participants who completed the survey and those who did not. However, the former were younger and had taken part in the teachers’ program in more recent years. These differences may be due to a technicality: Email records for participants who had completed the teaching program earlier had likely expired.

We reported the details of cultural distance calculation in the supplementary material.

We assessed inspiration with a 2-item measure. Participants rated the extent to which thinking about their years in the U.S. made them “feel inspired” and “inspired me to do something” (1 = *not at all*, 6 = *extremely*; *r* = 0.91, *p* < 0.001, *M* = 5.00, *SD* = 1.06). Although this measure has been used before^48,49^, we sought to re-validate it. In particular, we conducted a pilot study in which we examined convergent validity with a more established 8-item inspiration scale^17^. Fifty-two Amazon’s Mechanical Turk participants completed the brief measure and longer scale in random order. The two were highly correlated, *r*(50) = 0.76, *p* < 0.001.

We assessed intercultural exchange with a 3-item measure. Participants indicated their level of agreement with: “When I’m in my home country, I like to discuss issues related to the U.S. with my students or colleagues,” “When discussing issues related to the U.S., I tend to focus on the positive side of the issue,” “When I’m in my home country, I frequently mention my positive experience in the U.S. to my students or colleagues” *(1* = *strongly disagree*, 7 = *strongly agree*; α = 0.80, *M* = 5.96, *SD* = 1.02). The measure is related specifically to teacher experiences. Nevertheless, this operationalization maps onto our definition of intercultural exchange (“increase mutual understanding between people of two nations”), because it assesses teachers’ sharing of their host-country experiences with students and colleagues back home. Finally, we controlled for participants’ gender and age, as well as several variables that are likely to correlate with the extent to which repatriates felt inspired by U.S. culture and would engage in intercultural exchange (Supplementary Material).

### Study 2

We recruited as many participants as possible given budgetary and time constraints (*N* = 237; *M*_age_ = 36.14, *SD*_age_ = 7.69; 132 men, 105 women; 147 Caucasian, 30 African, 22 Latin American, 6 Native American, 8 Asian, 17 “mixed race” and 7 no responses). A sensitivity power analysis indicated that our sample size afforded sufficient power (0.80) to detect a small effect (*d* = 0.18).

We assessed acculturation using a 7-item Expatriate Adjustment Scale (^50^; − 3 = *unadjusted*, 0 = *neither adjusted nor unadjusted*, 3 = *adjusted*; α = 0.85). Next, we administered an 8-item scale to assess how inspired participants felt by the host country (17; 1 = *not at all*, 7 = *very deeply or strongly,* α = 0.95). We measured intercultural exchange with a single face-valid item: “How likely are you to recommend your friends from your home country to spend some time learning about [the host culture]” (− 3 = *definitely no*, 3 = *definitely yes*). A validation sample confirmed that this item converges with the 3-item scale that we used in Study 1 (more details of the measurement and validation study in Supplementary Material).

### Study 3

In Studies 1–2, we found that the effect of inspiration on intercultural exchange was large: the average correlation between inspiration and intercultural exchange was *r* = 0.56. However, here we erred on the side of caution and estimated a medium effect (*d* = *0.5*0). A priori power analysis indicated that a sample size of 128 would achieve sufficient power (0.80) and a sample size of 296 would afford high power (0.99). Given the inherent uncertainty surrounding the anticipated effect size, we recruited 300 participants to achieve high power and hedge against attrition. (*M*_age_ = 35.11, *SD*_age_ = 9.31; 143 men, 154 women, and 3 “Other”; 264 Caucasian, 22 Asian, 8 African/Caribbean, 2 Middle Eastern, 3 mixed race, and 1 “Other”).

In the inspiration condition, participants recalled and relived an experience of inspiration in the host country (“a time when you were inspired or experienced inspiration in [the host country]”). In the control condition, participants followed the same procedure, except that the target experience was representative of the participant’s daily life (“a specific experience that you feel is a good example of the kinds of experiences that you had in your everyday life in [the host country]”). The manipulation check comprised two items: “I felt inspired at those moments” and “Something inspired me” (1 = *strongly disagree*, 7 = *strongly agree; M* = 5.75, *SD* = 1.13; α = 0.93). We reported information on control variables and the coding of the intercultural exchange in the Supplementary Material.

### Supplementary Information

Supplementary Information.

## Data Availability

The data that support the findings of Study 1 are available from VIF International Education. Restrictions apply, as these data were used under license for the current study and so are not publicly available. However, the data are available from the authors upon request and with the permission of VIF International Education. The datasets (together with materials and codes) generated and analyzed for Studies 2 and 3 are available at: https://osf.io/nuxj6/?view_only=6c3641617469404e82394e9891642b55.
