# Supplementary material for: Cultural distances between home and host countries inspire sojourners to engage in intercultural exchange upon repatriation
Source: Sci Rep. 2023 Oct 16;13:17518. doi: 10.1038/s41598-023-44906-w (PMC10579379; doi:10.1038/s41598-023-44906-w)
Supplement: Supplementary file 1 — Supplementary Information. [file 41598_2023_44906_MOESM1_ESM.docx]

**Supplementary Material for**

**Cultural distances between home and host countries inspire sojourners to engage in intercultural exchange upon repatriation**

Xi Zou, ^1^* Dan J. Wang,^2^ Tim Wildschut,^3^ Constantine Sedikides,^3^ Dan Cable^4^

^1^ Nanyang Technological University, Singapore. https://orcid.org/0000-0001-6530-6076

^2^ Columbia University, USA

^3^ University of Southampton, UK

^4^ London Business School, UK

*To whom correspondence should be addressed.

**Email**: [zou.xi@ntu.edu.sg](mailto:zou.xi@ntu.edu.sg)

**This file includes:**

Supplementary text

Table S1

Table S2

**Study 1**

***Cultural Distance***

We derived cultural distance scores between a respondent’s home country and the U.S. using Hofstede’s four dimensions (i.e., cultural value orientation; 1): power distance, uncertainty avoidance, femininity/masculinity, and individualism/collectivism. We used the Kogut-Singh approach (4) to combine Hofstede’s dimensions into an overall index of cultural distance. To be precise, following more recent recommendations (5), we calculated the Mahalanobis (rather than Euclidean) distance between countries based on Hofstede’s dimensions. Each dimension was measured on a scale from 0 to 100. We based cultural distance scores on four of the six dimensions, because data for the remaining two dimensions (long-term/short-term orientation and restraint/indulgence) were unavailable for some countries in our sample. Among those countries for which we could calculate both 4-dimension and 6-dimension measures, the two measures were highly corrected (*r* = .94). Analyses based on the 6-dimension measure (capitalizing on a reduced sample) produced essentially identical results. We also calculated cultural distance using indices of the equivalent cultural dimensions from the GLOBE project (2) and Schwartz’s (3) value dimensions from World Values Survey, as well as a recently developed cultural distance index, which was moderately related to the Kogut-Singh Cultural Distance (*r* = .43, 4). All resulted in even greater attrition due to missing values, and therefore we did not adopt these measures. For example, three countries, Costa-Rica, Jamaica, and Venezuela were not included in the Muthukrishna et al. index (4), resulting in a reduction of more than 100 observations in the analyses.

As mentioned, instead of calculating the Euclidean distance between countries based on Hofstede’s dimensions (5), we calculated the Mahalanobis distance (6). Even though Euclidean and Mahalanobis distances are highly correlated (*r* = .90; 7), Mahalanobis distance is more appropriate because it accounts for the correlation between component variables, the different scales on which each variable is measured, and the variance of each variable. Accounting for collinearity between component variables is important for calculating cultural distance based on dimensions of cultural values, given that certain dimensions, such as power distance and individualism, are often highly correlated. Euclidean distance, for example, would weigh power distance and individualism equally even though they might represent the same underlying construct. In contrast, Mahalanobis distance uses information about the correlation between power distance and individualism to adjust the weight of each component in the distance calculation (6, pp. 1469-1470). In this sample, the country with the greatest cultural distance from the U.S. was Costa Rica (5.29), and the country with the smallest was Australia (0.39). The three most highly represented countries were Colombia (17.8% of respondents; cultural distance = 4.00), the U.K. (12.25% of respondents; cultural distance = 0.70), and Spain (8.1% of respondents; cultural distance = 2.69).

***Control Variables***

As in Study 1, we controlled for participants’ gender and age, as well as several variables that are likely to correlate with the extent to which repatriates feel inspired by U.S. culture and would engage in intercultural exchange.

**Years Since Return to Home Country.** Given that respondents who returned more recently might be more inspired and more likely to share ideas, we controlled for how long ago they returned. We calculated the number of years that had passed since participants returned to their home country (i.e., the year in which participants completed the present study minus the year of repatriation; *M* = 4.59, *SD* = 2.91).

**Total Years in the U.S*.*** The longer an individual remains in a host country, the more information they may be able to share upon returning, and the more inspired they may feel by their experiences abroad. We therefore calculated the number of years that participants stayed in the U.S. (i.e., the year of repatriation minus the year of program start; *M* = 3.13, *SD* = 1.97).

**Program Completion*.*** The most common reason participants reported for leaving their international teacher position was end of contract or visa expiration. Approximately 30% of the teachers left the program before the end of their contracts due to personal or family reasons, which could have been related to ease of repatriation. We created a dummy variable to differentiate participants who completed the international teacher program from those who did not (0 = *did not complete*, 1 = *completed*; *M* = 0.68, *SD* = 0.46).

**Big Five Personality*.*** The survey assessed the Big Five personality factors with Goldberg’s scale (8). Following the stem “I see myself as …”, participants responded (1 = *strongly disagree,* 7 = *strongly agree*) to the following traits: Conscientiousness (10 items; α = 0.69, *M* = 5.68, *SD* = 0.69), Agreeableness (9 items; α = 0.77, *M* = 6.06, *SD* = 0.63), Emotional Stability (9 items; α = 0.71, *M* = 4.83, *SD* = 0.81), Extraversion (10 items; α = 0.79, *M* = 5.20, *SD* = 0.86), and Openness to Experience (8 items; α = .52, *M* = 4.77 *SD* = 0.71). We included these five personality factors to control for potential self-selection bias.

**Country-Level Controls***.* Following the same logic that we presented in Study 1, we also controlled for political and economic factors that could be correlated with cultural distance. First, we used data from *The Economist*’s Democracy Index survey to control for political conditions favoring intercultural exchange. Also, we controlled for GDP per capita, total population, and immigrant population of respondents’ home countries, to account for demographic and economic features that might affect repatriates’ attitudes toward their experience working abroad.

***Alternative Mediation Model***

We compared the hypothesized mediation model to an alternative model in which the order of inspiration and intercultural exchange was reversed. Within a set of models for the same data, the Akaike Information Criterion (AIC; 9) can be used to compare competing models that need not be nested (smaller is better). However, any two models that have the same paths between the same variables will have the same fit, even if some paths are in a different direction. To allow a comparison between the hypothesized and alternative model, we therefore trimmed the direct effect of the predictor on the outcome variable in each model (i.e., the direct effect of cultural distance on intercultural exchange in the hypothesized model, and the direct effect of cultural distance on inspiration in the alternative model). The hypothesized model thus included two paths: one from cultural distance to inspiration, and one from inspiration to intercultural exchange (cultural distance ⇒ inspiration ⇒ intercultural exchange). The alternative model comprised a path from cultural distance to intercultural exchange, and a path from intercultural exchange to inspiration (cultural distance ⇒ intercultural exchange ⇒ inspiration). Results revealed a smaller (i.e., better) AIC value for the hypothesized (AIC = 4168.51) than the alternative (AIC = 4189.75) model. 4189.75) model.

***Additional Findings: Big Five Personality***

We did not formulate specific predictions regarding the associations of Big Five personality with inspiration and intercultural exchange, and few associations were significant in the regression analyses (see Table 2 in the main text). The exception was Agreeableness, which predicted increased inspiration and intercultural exchange. Agreeableness reflects the extent to which one is cooperative, friendly, generous, and unselfish (8). With the benefit of hindsight, it is perhaps not surprising that these characteristics would lead one to be inspired by a different culture and its people, as well as facilitate the social sharing of one’s intercultural experiences upon return to the home country. These findings offer additional construct validation for our measures of inspiration and intercultural exchange (10), and identify a fruitful direction for future research on the role of personality in this domain.

**Table S1.** Zero-Order Correlations Among Study 1 Variables

| Variables | 1 | 2 | 3 | 4 | 5 | 6 | 7 | 8 | 9 | 10 | 11 | 12 | 13 | 14 | 15 | 16 |
| --- | --- | --- | --- | --- | --- | --- | --- | --- | --- | --- | --- | --- | --- | --- | --- | --- |
| 1. Age | -- |  |  |  |  |  |  |  |  |  |  |  |  |  |  |  |
| 2. Sex (0 = man, 1 = woman) | -.01 | -- |  |  |  |  |  |  |  |  |  |  |  |  |  |  |
| 3. Years since return home | .21 | -.05 | -- |  |  |  |  |  |  |  |  |  |  |  |  |  |
| 4. Years abroad in U.S. | .15 | .00 | .02 | -- |  |  |  |  |  |  |  |  |  |  |  |  |
| 5. Program completion | .01 | .04 | -.04 | .31 | -- |  |  |  |  |  |  |  |  |  |  |  |
| 6. Extraversion | .11 | -.02 | .00 | .09 | .02 | -- |  |  |  |  |  |  |  |  |  |  |
| 7. Emotional Stability | .19 | -.09 | -.03 | .01 | -.00 | .30 | -- |  |  |  |  |  |  |  |  |  |
| 8. Conscientiousness | .15 | .07 | .01 | .11 | .04 | .34 | .36 | -- |  |  |  |  |  |  |  |  |
| 9. Agreeableness | .11 | .12 | .01 | .03 | -.01 | .39 | .32 | .59 | -- |  |  |  |  |  |  |  |
| 10. Openness to Experience | .02 | -.09 | -.02 | .03 | -.01 | .31 | .06 | .11 | .13 | -- |  |  |  |  |  |  |
| 11. Home country GDP per capita | -.10 | .06 | .18 | -.14 | -.12 | -.20 | -.16 | -.20 | -.17 | .01 | -- |  |  |  |  |  |
| 12. Home country population | .00 | .04 | -.01 | -.05 | .03 | .00 | -.02 | -.03 | -.01 | -.04 | -.13 | -- |  |  |  |  |
| 13. Home country immigrant pop. | -.10 | .01 | .19 | -.09 | -.07 | -.14 | -.16 | -.19 | -.12 | .05 | .75 | -.31 | -- |  |  |  |
| 14. Home country Democracy Index | .01 | .05 | .20 | -.11 | -.12 | -.16 | -.09 | -.09 | -.09 | .02 | .77 | -.27 | .60 | -- |  |  |
| 15. Cultural distance | -.04 | -.12 | -.16 | .17 | .06 | .20 | .12 | .11 | .10 | .03 | -.75 | -.03 | -.61 | -.67 | -- |  |
| 16. Inspiration | -.03 | -.06 | -.07 | .05 | .00 | .15 | .07 | .14 | .18 | .08 | -.28 | -.06 | -.17 | -.21 | .28 | -- |
| 17. Intercultural exchange | .07 | -.08 | -.08 | .10 | .00 | .22 | .10 | .22 | .30 | .08 | -.23 | -.07 | -.10 | -.17 | .23 | .46 |

*Note.* |*r|* ≥ .11, *p* < .001*;* .11 > |*r*| > .09, *p* < .01*;* .09 > *|r*| ≥ .07, *p* < .05

**Study 2**

***Expatriate Adjustment Scale***

We assessed acculturation with seven items from the Expatriate Adjustment Scale (11). Participants rated how unadjusted or adjusted they were when living in the host country on the following dimensions: living conditions in general; food; shopping; entertainment/recreation facilities and opportunities; socializing with host nationals; interacting with host nationals on a day-to-day basis; and speaking with host nationals (-3 = *unadjusted*, 0 = *neither adjusted nor unadjusted*, 3 = *adjusted*; α = .85).

***Inspiration***

Next, we administered an 8-item scale to assess how inspired participants felt by the host country (12)—the same scale that we used to validate the shortened version in Study 2. The 8-item scale is a condensed, face-valid measure of inspiration. It comprises a 4-item frequency subscale (1 = *never*, 7 = *very often*) and a 4-item intensity subscale (1 = *not at all*, 7 = *very deeply or strongly*) that are combined into an overall inspiration scale (α = .95). In our sample, the frequency and the intensity measures were highly correlated (*r*[237] = .86, *p* < .001). Thus, we combined these two dimensions into a single score.

***Intercultural Exchange***

We measured intercultural exchange by instructing participants to rate a single face-valid item: “How likely are you to recommend your friends from your home country to spend some time learning about [the host culture]” (-3 = *definitely no*, 3 = *definitely yes*). Single-item measures are suitable when, as in our case, survey space is limited and the question is “very concrete and it can be reasonably assumed that there is virtually unanimous agreement among respondents as to what characteristic is being measured” (13, p. 446).

Notably, this measurement is different from study 1. Whereas this methodological diversity strengthens convergent validity (14), it is important to verify that the various measures indeed reflect the same underlying construct. For this purpose, we conducted a validation study in which we administered the complete set of self-report measures and ended the survey with questions on participants’ demographics. We recruited through Qualtrics 85 repatriates (*M*_age_ = 35.91, *SD*_age_ = 7.80; 59 men, 26 women; 61 Caucasian, 3 Latin American, 2 Asian, 10 African, 1 Middle Easter, and 8 Native American). They met the following criteria: (1) American citizens born and raised in the U.S.; (2) lived and worked in a foreign country for over six months; (3) returned to the U.S. from the foreign country within the last five years; and (4) currently employed full-time in the U.S. Most participants had lived and worked in European host countries (44 in the U.K., 28 in Germany, four in Spain, two in Ireland, two in Scandinavia), although four had lived and worked in Australia and two in Hong Kong.

In Study 1, intercultural exchange was measured by three items: “When I’m in my home country, I like to discuss issues related to the [host country] with my colleagues”; “When discussing issues related to the [host country], I tend to focus on the positive side of the issue”; and “When I’m in my home country, I frequently mention my positive experience in [host country] to my colleagues” (1 = *strongly disagree*, 7 = *strongly agree*; items 2-4). We present descriptive statistics and correlations among these four items in Table S1. We conducted a principle axis exploratory factor analysis. All four items loaded onto one factor (Eigenvalue = 2.10, accounted 54% of the variance). Respective factor loadings for items 1-4 were: .70, .83, .87, and .50.

**Table S2.** Zero-Order Correlations Among Self-Report Items Assessing Intercultural Exchange in Validation Study Validation Study

|  |  |  | Correlations | | |
| --- | --- | --- | --- | --- | --- |
|  | *M* | *SD* | Item 1 | Item 2 | Item 3 |
| Item 1 | 6.22 | 0.79 |  |  |  |
| Item 2 | 6.01 | 1.04 | .40*** |  |  |
| Item 3 | 5.85 | 1.14 | .45*** | .68*** |  |
| Item 4 | 2.38 | 0.87 | .22* | .22* | .30** |

*Note.* Item 1: “When I’m in my home country, I like to discuss issues related to the [host country] with my colleagues.” Item 2: “When discussing issues related to the [host country], I tend to focus on the positive side of the issue.” Item 3: “When I'm in my home country, I frequently mention my positive experience in [host country] to my colleagues” (1 = *strongly disagree*, 7 = *strongly agree*; items 1-3). Item 4: “How likely would you recommend your friends from your own country to spend some time to learn about [host country]’s culture?” (-3 = *definitely no*, 3 = *definitely yes*).

* *p* < .05, ** *p <* .01, *** *p* ≤ .001

**Study 3**

**Information on Control Variables**

***Positive Affect***

We measured positive affect using four items (“excited,” “enthusiastic,” “alert,” “determined”) from a short version of the Positive and Negative Affect Schedule (PANAS, 15; 1 = *very slightly*, 7 = *extremely; M* = 4.98, *SD* = 1.23; α = .86). The initial version of the PANAS included a fifth item, “inspiration,” but we excluded it because it was part of the manipulation check.

***Negative Affect***

We measured negative affect using five items (“upset,” “distressed,” “scared,” “nervous,” “afraid”) from the short PANAS version (15; 1 = *very slightly*, 7 = *extremely; M* = 1.42, *SD* = 0.84; α = .92).

***Effort***

We used two items (16) to assess effort during the essay writing: “I was working hard” and “I was putting forth a great deal of effort” (1 = *strongly disagree*, 7 = *strongly agree*; *M* = 5.25, *SD* = 1.09; α = .88).

**Intercultural Exchange**

We recruited two coders who were blind to the experiment’s hypothesis and presented them with the participants’ essays. We informed the coders that each essay describes a foreign country, and their task was to rate the extent to which each essay “makes you want to visit this foreign country” (1= *not at all*, 2 = *slightly*, 3 = *moderately*, 4 = *very much*; Spearman-Brown interrater reliability = .88). We averaged the coders’ ratings to form a composite index of intercultural exchange.

**References**

1. G. Hofstede, *Culture’s consequences: Comparing values, behaviors, institutions, and organizations across nations*, Sage Publications (2001).
2. R. Maseland and A. van Hoorn, *Explaining the negative correlation between values and practices: A note on the Hofstede-GLOBE debate*, J. Int. Bus. Stud. **40**(3), 527-532 (2009).
3. S. H. Schwartz, *Are there universal aspects in the structure and contents of human values?* J. Soc. Issues. **50**(4), 19-45 (1994).
4. M. Muthukrishna, A. V. Bell, J Henrich, J., C. M. Curtin, A. Gedranovich, J. McInerney, and B. Thue, Beyond Western, Educated, Industrial, Rich, and Democratic (WEIRD) Psychology: Measuring and Mapping Scales of Cultural and Psychological Distance. *Psychological Science*, 31(6), 678-701 (2020).
5. B. Kogut and H. Singh, *The effect of national culture on the choice of entry mode*, J. Int. Bus. Stud. **19**(3), 411-432 (1988).
6. H. Berry, M. F. Guillén, and N. Zhou, *An institutional approach to cross-national distance*, J. Int. Bus. Stud. **41**(9), 1460-1480 (2010).
7. I. R. P. Cuypers, G. Ertug, P. P. M. A. R. Heugens, B. Kogut, and T. Zou, *The making of a construct: Lessons from 30 years of the Kogut and Singh cultural distance index*, J. Int. Bus. Stud. **49**(9), 1138-1153 (2018).
8. L. R. Goldberg, L. R. *The development of markers for the Big-Five factor structure*, *Psycho Ass,* **4**(1), 26-42 (1992).
9. H. Akaike, *A new look at the statistical model identification.* IEEE Transactions on Auto Cont, *19*(6), 716-723 (1974).
10. L. J. Cronbach and P. E. Meehl, Construct validity in psychological tests. *Psy Bul,* **52**(4), 281-302 (1955).
11. J. S. Black and G. K. Stephens, *The influence of the spouse on American expatriate adjustment and intent to stay in Pacific Rim overseas assignments*. *J Mana,* **15**(4), 529-544 (1989).
12. T. M. Thrash and A. J. Elliot, *Inspiration as a psychological construct*, J. Pers. Soc. Psychol. **84**(4), 871-889 (2003).
13. A. Diamantopoulos, M. Sarstedt, C. Fuchs, P. Wilczynski, and S. Kaiser. *Guidelines for choosing between multi-item and single-item scales for construct measurement: A predictive validity perspective.*J. Acad of Mark Scie*, 40*(3), 434-449 (2012).
14. D. T. Campbell and D. W. Fiske. *Convergent and discriminant validation by the multitrait-multimethod matrix*. Psy Bull*,* **56**(2), 81-105. (1959).
15. K. Kercher, K. *Assessing subjective well-being in the old-old: The PANAS as a measure of orthogonal dimensions of positive and negative affect.* Rese on Agin, **14**(2), 131 (1992).
16. T. M. Thrash, L. A. Maruskin, E. G. Moldovan, V. C. Oleynick, and W. C. Belzak. “Writer–Reader Contagion of Inspiration and Related States: Conditional Process Analyses Within a Cross-Classified Writer X Reader Framework,” J. Pers. Soc. Psychol. **113**(3), 466–91 (2017).
